# Valorization of Food Waste as Animal Feed: A Step towards Sustainable Food Waste Management and Circular Bioeconomy

**DOI:** 10.3390/ani13081366

**Published:** 2023-04-16

**Authors:** Pinku Chandra Nath, Amiya Ojha, Shubhankar Debnath, Minaxi Sharma, Prakash Kumar Nayak, Kandi Sridhar, Baskaran Stephen Inbaraj

**Affiliations:** 1 Department of Bio Engineering, National Institute of Technology Agartala, Jirania 799046, India; 2 Department of Applied Biology, University of Science and Technology Meghalaya, Baridua 793101, India; 3 Department of Food Engineering and Technology, Central Institute of Technology Kokrajhar, Kokrajhar 783370, India; pk.nayak@cit.ac.in; 4 Department of Food Technology, Karpagam Academy of Higher Education (Deemed to Be University), Coimbatore 641021, India; 5 Department of Food Science, Fu Jen Catholic University, New Taipei City 242062, Taiwan

**Keywords:** food waste (FW), animal feed, recycling, waste management, treatment technology

## Abstract

**Simple Summary:**

Have you ever thrown away food that you didn’t eat? Most people do this all around the world but throwing away food can actually harm the environment. One way to reduce this harm is by turning the food scraps into animal feed. This not only helps the environment but also makes livestock production cheaper. Different technologies have been developed to make a safe and healthy animal feed from food waste. This helps us to get rid of waste by giving animals a new source of protein and recycling the discarded food waste. This article talks about how to turn food waste into animal food and its advantages. However, it is important to make sure the feed is of high quality and safe for the animals. It is also important to do research and development to make even better food-waste-based animal feed by reducing production costs and waste disposal, thereby making things better for both the animals and the environment. Overall, using food waste as animal food is a good waste management idea that provides food security and preserves the environment. So, next time when you have some leftover food, remember that it could be turned into something useful instead of being thrown away.

**Abstract:**

The growing population and healthy food demands have led to a rise in food waste generation, causing severe environmental and economic impacts. However, food waste (FW) can be converted into sustainable animal feed, reducing waste disposal and providing an alternative protein source for animals. The utilization of FW as animal feed presents a solution that not only tackles challenges pertaining to FW management and food security but also lessens the demand for the development of traditional feed, which is an endeavour that is both resource and environmentally intensive in nature. Moreover, this approach can also contribute to the circular economy by creating a closed-loop system that reduces the use of natural resources and minimizes environmental pollution. Therefore, this review discusses the characteristics and types of FW, as well as advanced treatment methods that can be used to recycle FW into high-quality animal feed and its limitations, as well as the benefits and drawbacks of using FW as animal feed. Finally, the review concludes that utilization of FW as animal feed can provide a sustainable solution for FW management, food security, preserving resources, reducing environmental impacts, and contributing to the circular bioeconomy.

## 1. Introduction

Food waste (FW) is described as the loss of food that occurs at the end of the food chain. This loss of food results in a loss of resources such as labour, water, energy, and land that were used in production, as well as losses for retailers and customers [1]. Exorbitant amounts of food are wasted worldwide as a result of differences in food production, transportation, and consumption [2]. Compounding issues include the ongoing creation of garbage and the concurrent migration of people from rural to urban areas. Because of the variety in generation patterns, chemical and physical characteristics, as well as underlying challenges and differences in assessing their growing volume, managing food loss and waste is a huge task [3]. By 2050, researchers estimate that 68% of the world’s population will reside in urban areas, leaving only 30% of the population to produce the vast quantities of fruits, vegetables, and animal products needed by urban dwellers [4]. The features and composition of wasted food in various studies have also been summarized in the process of evaluating the policies and treatment alternatives in light of their significance in choosing the best prevention policies and treatment approaches [5]. Whereas the latter has been explored in terms of the recovery (reuses and recycle) and disposal of the FW hierarchy, the former has been illustrated via an analysis of the policies and regulation systems that have been enacted [6]. This has been utilized to gain understanding of the motivations guiding initiatives to handle FW sustainably [7]. “Circular economy” and “bioeconomy” are concepts on the cutting edge of change. According to the Circular Economy Action Plan [8], “circular economy” is defined as the long-term conservation of product value, materials, and resources in the economy with reduced waste generation. FW, in particular, is an important aspect of the circular economy and should be considered at various levels throughout the value chain. Food that is excreted and digested ends up as organic waste, energy recovery, or landfill disposal. Reduction of food loss and waste is a serious challenge in India, which needs to feed its rapidly growing population (1.7 billion by 2050) [9]. In order to alleviate the environmental burden that is caused by FW, alternative methods are required to repurpose FW into uses with a higher value [10,11]. This will minimize the impact that FW has on the environment and promote the long-term sustainability of our system of obtaining food [12]. The utilization of FW as an alternative source of animal feed contains a significant amount of promise for the purpose of overcoming the existing precarious situation, which is characterized by excessive costs and an inadequate supply of livestock feed [13,14]. This review discusses a complete explanation of the FWs and types, nutritive attributes of FWs, meat quality and animal growth, energy consumed to produce animal feed, types of FWs for animal feed, and the methods of converting FWs into animal feed, as well as the limitations of this process and the benefits and drawbacks of using FWs as animal feed.

## 2. Food Waste and Types

**Figure 1:**
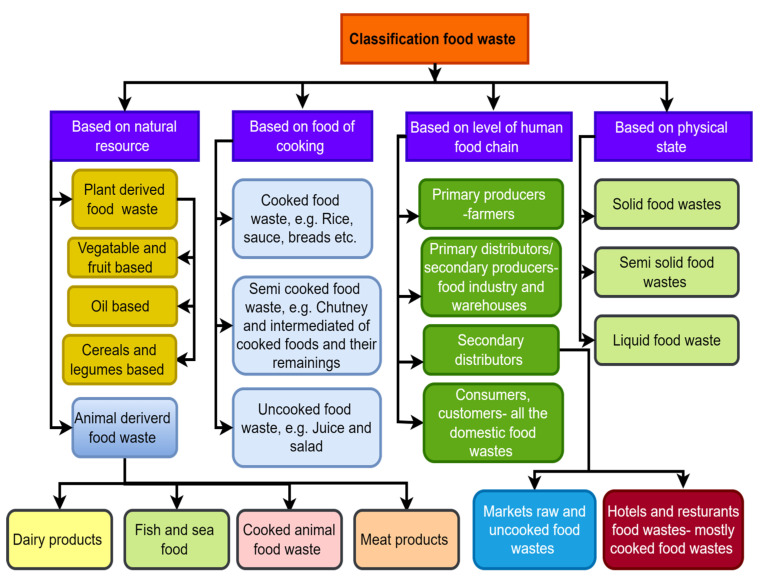
Overview of classification of FW.

Depending on its nature and origin, FW can be categorized in a variety of ways. On the basis of their primary natural resource, FW can be classified as either plant or animal derived [15]. Similarly, FW may be classed as raw or uncooked FW, cooked FW, or semi-cooked FW, depending on the cooking method. Food is transported from the producer to the customer via a variety of routes during the production process, and waste is created at each of these levels. Agriculture is the first link in the food chain. As a result, growers are regarded as the main producers. At the secondary level of the human food chain, warehouses and businesses such as flour mills and food preservation mills can be thought of as primary distributors [16]. Due to the fact that they create processed food ingredients, they might also be considered secondary producers [17]. Markets, hotels, and restaurants are categorized as secondary and direct distributors at the third level of the human food chain [18]. Customers (also known as consumers) represent the apex of the food chain. Based on this human food chain, FW can be classified as follows: (i) agro FWs—that comes from farmers, (ii) industrial FWs—generated by wholesalers, (iii) market FWs—fresh and uncooked food made by secondary distributors, (iv) hotel and restaurant FWs—additionally generated by secondary distributors in the form of cooked products, and (v) domestic FWs—generated by customers. FW is divided into three types based on its physical state: solid FWs, semisolid FWs, and liquid FWs. Figure 1 depicts the overall classification of FWs.

## 3. Nutritive Attributes of FWs

The nutritional value of FW or loss per day has been estimated to be approximately 1200–1500 food calories [19]. Carbohydrates make up around 30 to 60% of FWs, whereas proteins range from 5 to 10%, and fats make up 10 to 40% [20]. The nutrients that are present in foods that are discarded are lost when those items are wasted. The generation of FW, measured in grammes per person per day, can be broken down into the following categories: cooked food (56%), vegetables (18%), fruits (16%), dairy (3%), and cereals (4%) [21]. Most food waste is a good source of nutrients for animals to eat. Therefore, FWs can be used as an alternative to feed for animals. It is thought that 1 tonne of dry FW could be used instead of the same amount of maize grain to meet an animal’s protein needs [22]. FW can be used instead of maize, which is a main feed source and has 8 to 10 percent protein [23]. Organic waste is being composted with the help of insects, which is a new trend. Insects are also being used as animal food because they are more nutritious than other foods. The fact that a mature larva of the black soldier fly *Hermetiaillucens* has 40 to 45% protein in biomass and up to 35 percent fat by dry weight demonstrates its usefulness as animal feed [24]. A study on *H. illucens* demonstrated that the fly can ingest a variety of organic wastes, including poultry feed, pig liver, pig manure, kitchen trash, vegetables and fruits, and rendered fish, with kitchen waste exhibiting the highest fly biomass output [24].The concentration of bioactive chemicals and polyphenols in food waste peel, pomace, and seeds is double that of the edible component used in animal feed production. The chemicals found in FW have anti-cancer, anti-bacterial, anti-oxidative, and immune-stimulant properties in vertebrates, as well as being linked to a lower prevalence of cardiovascular disease [25].FW has dyes such as carotenoids from tomato peel and carrot pomace, anthocyanin from banana bracts, and betalains from beetroot pulp. These dyes have anti-oxidant activities that can be used to protect living systems from oxidative damage by removing oxygen free radicals and making foods more stable by preventing lipid peroxidation in animals [25].

## 4. Food Waste in Animal Feed Production

Three times as much food is wasted as is produced each year (or 1.3 billion tonnes) [26]. “Food waste” is commonly used to describe the phenomenon of food spoilage in the last phases of the food supply chain, including retail and final consumption. “Food loss” is the term used when food is wasted during the production, post-harvest, and processing phases of the food supply chain. As one of the most underutilized resources, micronutrients in food are lost at an alarming rate due to FW. ”Food waste” is the term for wasting ideal nutrients meant for human consumption, which normally have good nutritional value. From the early and intermediate stages of the food supply chain, losses in impoverished nations substantially outweigh the FW that occurs in retail and ultimate consumption, it may be concluded that the bulk of food spoilage occurs during manufacture, transit, and warehousing [27]. Despite the importance of decreasing FW, the circular economy, which sees waste as a valuable resource, calls for the reintroduction of food scraps into the food supply chain [28].

Scientists from around the country (Table 1) are largely working on reintroducing food waste and surplus vegetables and fruits into the food chain as animal feed [29]. Instead of feed grains or protein sources, livestock ranchers have historically fed their animal’s food scraps [30]. Farmers can increase their earnings by utilizing food scraps to lower the cost of animal feed. Another key advantage is the reduction of environmental difficulties caused by the decomposition of such wastes. The use of industrial FW as animal feed comes with a number of drawbacks in addition to the benefits that were discussed before. These drawbacks include a lack of safety, an unpredictable nutrient profile, and expensive production costs. Because they include a high percentage of water, food scraps are more likely to deteriorate during the collection, transportation, and storage processes. As a consequence of this, the quality of animal feed that is manufactured from FW suffers during the processes of garbage collection, transportation, and storage [31]. In addition, commercial FW (namely waste from the food service and retail sectors) varies in both quantity and homogeneity and its nutritional make-up is inconsistent [32]. Manufacturers of recycled animal feed have addressed these challenges with innovative thinking and now successfully recycle FW into animal feed at a relatively low cost [33].

Particle solids from the waste solids separation process can be used in animal feed. These items can be used to dry or pellet FW for sale as animal feed. Animal feed has long been used and will continue to be utilized in the production of meat and poultry. Animal feed frequently contains blood, feathers, and bones that have been processed into a meal. Leftover meat that is deemed unfit for human eating is either sold to rendering enterprises or sent directly to them in order to prepare animal and pet meals [53]. The use of these resources benefits both the environment and the economy. This is due to the way they are marketed in comparison with other fats, vegetable oils, and proteins.

It has been common practice for many years to incorporate food scraps and other forms of trash into the diets of animals, particularly those that are kept on farms [54]. According to the FAO, around 30 percent of all of the world’s cow feed is comprised of waste or leftovers from the production and processing of food goods [55]. However, a sizeable amount of freshly produced food might be better suited for use as animal feed rather than being disposed of in landfills, where it would add to the greenhouse effect by releasing methane [56]. Landfills are one of the most significant contributors to the problem [57].This would be the environmentally preferable alternative. Reusing nutrients for feed through more circular systems can also assist to reduce the significant environmental impacts of growing feed crops, such as the consumption of land, energy, and water, while simultaneously improving food security by reducing FW and increasing food production [58]. Despite its apparent simplicity, returning FW into the food chain presents numerous obstacles. The ideal feed option for livestock must always be available and affordable. The pros and cons of reintroducing this waste into the food supply chain, as well as the environmental consequences, must also be considered [59]. Converting “waste into development potential” can be an effective strategy for preserving sustainability in the livestock industry [60].

According to Moult et al. [61], people think about environmental effects other than greenhouse gas emissions when they think about the possibility of using food waste as animal feed. Retailers’ choices regarding the handling of food waste are heavily influenced by considerations of their effect on the environment. Greenhouse gas (GHG) production, water consumption, and contamination of water, air, and soil systems are all examples [62]. Any way of getting rid of food waste will have a different carbon footprint depending on what kind of food is being thrown away and how it is thrown away. The average amount of methane captured by landfills around the world is 20% (landfills with no gas collection equipment) [63]. Due to the rules stated above in the UK, some of these ways to get rid of food are fictional, such as turning it into raw meat or feeding it to animals. However, they are still included in the list of GHG emissions so that the full picture can be seen.

During the manufacturing process, food waste can be generated from products that are damaged or of poor value that are abandoned in the field. Damage to the food while it is being transported, deterioration or contamination with bacteria while it is being stored, and losses while it is being processed all contribute to the wastage of food [64]. Food is thrown away in the retail system as a result of handling-related damage, a dearth of cold storage, and inadequate inventory management. Overbuying, improper storing, excessive preparation, improper portioning or heating, and inadequate reading of product labels are the primary causes of food waste produced by individual consumers [65]. An increased risk of infection occurs in a cyclical food system due to the retention and accumulation of microbial pathogens.

## 5. Methods of Converting FWs into Animal Feed

**Figure 2:**
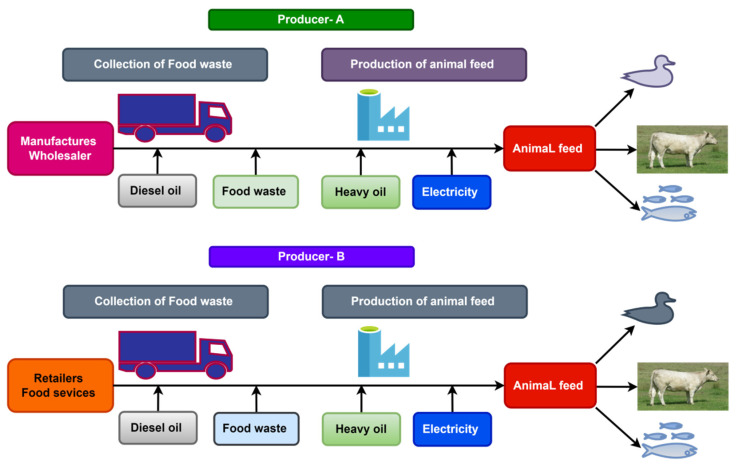
Utilization of FWs to animal feeding by producers (**A**,**B**).

A range of processing processes are used to increase the nutritional content, digestibility, feeding efficiency, removal of toxins, pathogen sanitation, removal of non-edible components, feasibility for long-term storage, transportability, and marketability of FW [66]. FWs converting to a value-added product, such as animal feed, can enhance food efficiency by lowering the cost of animal feed, resulting in higher profitability for farmers and decreased environmental consequences caused by FW disposal [67]. Processing foods by changing their physical (and seldom chemical) qualities is a crucial stage in any such conversion process to improve feed quality, stable feed in the animal diet, and decrease loss during feeding [68]. The methods for processing FW concentrate primarily on feed conversion efficiency, higher feed intake, and cattle health, with decreased digestive diseases. Several processing techniques, including dehydration and/or drying, pelleting, extrusion, fermentation, silage production, etc., can be utilized to convert food scraps into animal feed. In order to transform a certain type of FW into an acceptable animal feed, these processing technologies are either combined or used independently. Conversion of FWs into animal feed by each producer is shown in Figure 2.

### 5.1. Solar Drying

**Figure 3:**
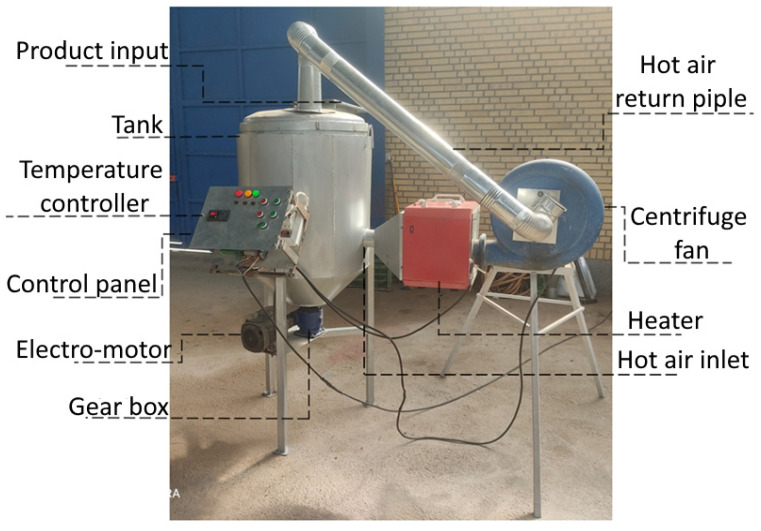
Components of waste dryer. Figure 3 reprinted with permission (copyright © 2022, Elsevier Ltd., Amsterdam, The Netherlands) from Rahmani et al. [69].

The Gorgan University of Agricultural Sciences and Natural Resources in Iran developed and produced a waste dryer with a 25 kg capacity [69]. Figure 3 depicts the device’s major components. According to Bulgakov et al. [70] a vibrating dryer for FWs was simulated. Their theoretical studies have revealed that this form of drier is able to minimize humidity as a guarantee. Song et al. [71] also created a food waste dryer. If water is evaporated by the drier, the drying rate will be 19.65 percent, and if a mixture of water and other substances is dried, the rate will be 3.85 percent. In order to create animal feed, Rahmani et al. [69] studied the energy and exergy of semi-industrial FW drying equipment. The amount of energy that is being used, as well as the amount of lost exergy and the rate at which the potential is being improved, grows as the temperature rises [69]. The amount of energy needed to make feed from FW is 18.30 MJ/kg, which is 1.8 MJ/kg less than the amount of energy needed to make feed from corn [69]. Producing animal feed from FW is a viable option because it requires less resources and costs less money compared with other feed sources [69].

### 5.2. Spray Drying

**Figure 4:**
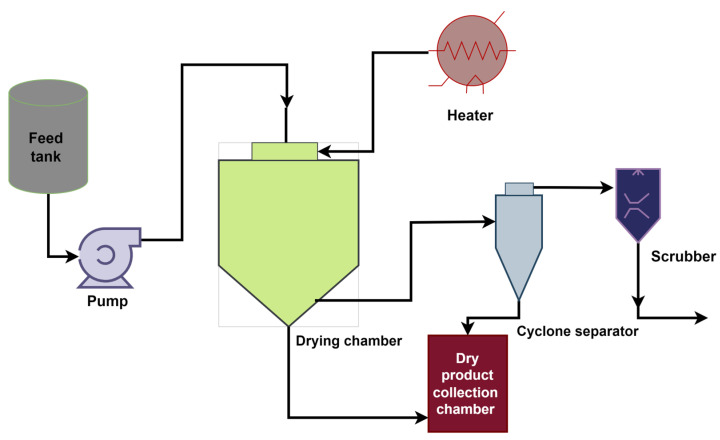
Spray dryer working procedure.

Spray drying is one of the simplest ways to extend the shelf life of liquid extracts and improve the organoleptic features of products by converting extracts into a stable dry powder (Figure 4). With spray drying, liquid feeds (extracts) are atomized into the drying chamber, then the resulting droplets pass through a hot-air (or sometimes nitrogen) stream to evaporate [72]. The evaporation of water takes place at a faster pace when the droplets themselves are smaller since this increases the surface-to-mass ratio. Because of the speed and intensity with which this procedure is carried out, there is very little heat damage caused to sensitive material.

Several studies have employed microencapsulation by spray drying to create anthocyanin concentrates from sources such as grapes, cranberries, Roselle black carrots, hibiscus extracts, blueberry by-products, blackcurrants, bayberry juice, and many more [73,74]. An enormous amount of waste and by-products are produced during the processing of the plants used in the food, beverage, cosmetic, and pharmaceutical industries [75]. Peels, husks, unused plant components (seeds, roots, and broken leaves), and oil seed cakes are a few examples of plant by-products. Some non-plant businesses, such asthe fish and meat sectors, also produce waste and by-products. According to the FAO, without retail and consumer generated waste, 13.7% of all food is discharged as by-products and waste [76]. On the other hand, Gustavsson et al. [77] found that a third of the weight of all the food produced globally is regarded as waste. According to Red Corn et al. [78], over a quarter of all edible food is wasted. The phrases most frequently used to define losses during production, post-harvesting, and processing are “food wastes”, “food losses”, and “waste of plant origin”. Numbers are inconsistent and widely variable due to reporting differences; however, anywhere between 6% and 25% of the total amount of food produced is thrown away as waste [79]. One of the key objectives for sustainable development is the reduction and reuse of FW. Several sectors suffer large financial losses as a result of underutilizing by-products. Additionally, producers typically have to cover the cost of waste transportation and disposal [80].

### 5.3. Dehydration

For the management of home FW and pre-treatment using dehydration of FW segregated at source, the study’s methodology involved the use of a cutting-edge household garbage dryer. In this study, a novel approach for the separation and dehydration of FW separated at source is presented together with the methodology and findings of the first pilot-scale demonstration [81].The sanitary dehydration of FW at the source resulted in a large mass reduction, approximately 70% *w*/*w*, due to the removal of moisture content throughout the drying process, while the energy requirements remained economically acceptable [82].The created biomass’s low water content avoids biological decomposition, limits odour emissions, and so minimizes the frequency with which residential trash must be collected. Additionally, dry biomass is considerably easier to handle than wet FW. Moreover, the waste can be used in environmentally friendly and alternative methods to create high-value goods such as compost, bioethanol, biogas, animal feed, and thermal energy [83]. This is demonstrated by the waste’s evaluated physicochemical properties. Table 2 lists certain FWs and their basic processing techniques for the production of animal feed.

### 5.4. Freeze Drying

High-quality fruits and vegetables that have been dehydrated are frequently processed via freeze-drying. During freeze-drying protection, the water solid-state, low temperatures, and moisture sublimation processes preserve the fundamental structure and shape of the products, which also have a low bulk density, high porosity, and a stronger rehydration.

To remove the unfrozen solvent from a liquid formulation solvent, a desorption process must be used after the solvent has been extracted, frozen, and subjected to low pressure to cause the solvents to sublimate [105]. As a result, the process of drying can be broken down into two stages: sublimation (also known as primary drying) and desorption (also known as secondary drying). This is because both stages involve two processes that are equally as significant: freezing, in which almost all of the solvent is transformed into a solid that is frozen, and drying, in which the mixture almost completely gets rid of all of the solvent (frozen or unfrozen) [106].

In the process of freeze-drying, the food is first frozen, which consolidates it. The size and development of ice crystals depend on the freezing rate; sluggish freezing results in larger or smaller ice particles [107]. Only high-value foods such as coffee, ingredients for ready-to-eat meals (fruits and vegetables or meat and fish), and aromatic herbs are typically freeze-dried in the food industry. The solid waste produced during lengthy human-crewed space trips has been extensively handled by the MEADOW processor freeze-drying solid waste [108]. The two main drying techniques investigated were freeze-drying and vacuum-drying. A Peltier condenser collects wastewater vapour in either mode or transforms it back into relatively pure water. The dried waste product has less water activity than what is necessary for bacteria to continue their metabolic activity. The treated waste must be contained and kept in a way that prevents water from being reabsorbed for it to be stable. Many studies have shown that freeze-drying is an effective method for minimizing FWs [109]. However, they argued that freeze-drying was an inappropriate way to dispose of garbage due to its high cost. Yet, the unique circumstances will aid in minimizing waste for animal feed [110].

### 5.5. Microwave Drying

Processing agricultural crops after harvest effectively uses microwave drying. The drying sector can benefit greatly from three main factors: its speed, low energy usage, and good product quality [111]. The development of new technologies has made it possible to monitor and manage an increased number of parameters during the drying process. These characteristics include temperature, weight, power, aroma, and others. According to Bai et al. [112], the increased relative humidity of convection air may be a significant component that slows drying while preserving the quality of the products’ interior or exterior surfaces. The volatile compounds that are continuously released from the material being dried during microwave drying are transported by convection air. The moisture transport equation, whose driving force depends on the concentration difference, is comparable to the diffusion model for volatile chemicals [113]. There is very little research on using high humidity throughout the drying process [114]. Moreover, these works solely used natural, sun, and convective drying processes; microwave drying was not used. Microwave drying, which could be a valuable technique for processes such as drying vegetables and fruits in closed packages, requires research on the impact of high humidity on the drying rate as well as the product quality [115]. In this study, an effort is made to track and manage the convective air’s humidity in an effort to solve the issue. For this, a novel microwave drying technology was created. The system included three subsystems: a one-way air flow control system for removing the gaseous moisture, a thermostatic microwave heating system with online mass weighting, and a humidity measuring system [116]. Since waste foods are simple to obtain and have highly volatile chemicals, they were chosen as the drying samples. For the objective of changing the relative humidity surrounding the samples, various plans were made. Analysis and discussion were conducted on the connections between relative humidity and drying speed as well as between relative humidity and product quality [117]. The findings will be used to support the claim that high humidity improves the quality of the produced products.

### 5.6. Silage

Agriculture has undergone significant changes since the 1960s, with a focus on using science to produce crops, increased mechanization, and larger livestock farms [118]. With better animal diet and genetics, there was an increase in output per head of livestock. Between 1975 and 2000, the production of silage dry matter (DM) increased across the majority of European nations [119]. A decrease in hay output and an increase in the size of animal farms over that time period both contributed to this in part. In contrast, hay production increased substantially in the US during the same time period, with crops such aslucerne being cultivated expressly for that purpose [120]. The US had a similar pattern.

Farmers can employ grains and other commercial feeds, conserve seasonally surplus grass or other fodder crops, or a combination of these methods to manage seasonal feed shortages. Before to the 1960s, fodder was primarily stored as hay, typically gathered at a mature growth stage and vulnerable to weather uncertainties throughout the lengthy intervals between cutting and harvest [119]. This definition of silage refers to the collection and storage of fermented materials such as fermented whole-crop cereals, fermented crop by-products, and moist grass for use as livestock feed. Crop production, engineering, chemistry and biochemistry, microbiology, and animal nutrition are a few of the scientific and technological fields that are involved in the conservation process [121]. Because of this, the successful production of silage necessitates an understanding of the critical physical, chemical, and biological elements affecting the entire conservation process, among which oxygen and water are the most crucial, at least in terms of nutrient losses. Silage plays two crucial roles in the nutrition of livestock: (i) it serves as a preserved source of digestible nutrients in diets for high-producing animals to maintain optimal rumen function and lowers the risk of diseases such as rumen acidosis and displaced abomasa; and (ii) it serves as a supplemental feed to be used when the rate of pasture growth is insufficient in relation to animal needs, such as in the winter and during dry spells [122,123].

## 6. Meat Quality and Animal Growth

The proportion of FW that was employed in meals (the replacement rate) ranged from 10% all the way up to 100% in the various feeding experiments that were conducted [124]. Responses in terms of animal weight increase and/or feed use efficiency vary based on the animal species and physiological stage, as well as the length of the feeding trial, the type of FW, and the substitution rate [125]. A number of studies found no difference when comparing diets with substitution with diets without substitution [126,127,128]. On the other hand, other studies found that diets with substitution caused poultry [129] and pigs [130] to gain less weight. The findings of various investigations were compiled by Guo et al. [131], who found that pigs with a substitution rate of 50% had a growth rate that was 13% slower.

The quality of meat has been studied by comparing diets with and without food waste [132]. For instance, Katajajuuri et al. (1998) [133] discovered that pork from animals given heat-treated FW was comparable in flavour and quality with meat from pigs fed a maize–soy diet, as rated by a panel of volunteers. Giamouri et al. [134] revealed that, through blind tasting, panellists preferred the softness of lean meat from pigs fed a diet of liquid food waste over that of animals fed a regular diet. Using linear mixed models to examine the impacts of the inclusion of FW in pig diets, with the original data gathered from many studies, Giamouri et al. [135] found that feeding FW had no impact on 16 of 18 pork quality measures (e.g., juiciness, dressing percentage, meat colour, fat-free lean percentage, flavour, overall palatability, etc.). The discovered effects of two criteria (monounsaturated fats and marbling) were “weak and did not detrimentally affect pork quality or value”. The researchers concluded that including food waste into diets resulted in pork of comparable quality with that generated by animals fed normal diets [135].

## 7. Energy Consumed to Produce Animal Feed

Corn is one of the most commonly utilized products for animal feed. To determine the energy (Equation(1)) required for manufacturing animal feed from corn, it is necessary to compute all the energy entering the farm per kg of corn. Livestock production rises by an average of 2.46% each year [136], whereas the demand will rise owing to population expansion. Many sources (fossil energy, water, and land) are utilized to generate livestock, poultry, and aquatic feed, but the energy required for maize production in Iran comprises machinery, human labour, seeds, fertilizers, chemicals, and water. According to Banacian and Zangench [137], each kg of corn requires an average of 7.24 MJ of energy to grow. Similarly, Rahmani et al. [69] assumed that transporting maize to the feed mill consumed 0.4 MJ per kg of corn energy. Furthermore, the energy required for the conversion of corn-to corn guten feed has been calculated to be 12.46 MJ/Kg. Eventually, the production of each kg of livestock feed is calculated to be 20.10 MJ per kg.

(1) $$EU_{CM}=\sum EU_{IF}+EU_{TR}+EU_{PR}$$

where $EU_{CM}$ is the energy consumed to produce animal feed, *EU*_*IF*_ is the total energy input to the farm, $EU_{TR}$ is the energy consumption for transportation, and $EU_{PR}$ is the total energy consumption for processing.

## 8. Types of FWs to Animal Feeds

A wide variety of domestic animals receive their nutrition from a variety of food scraps and waste products of the food industry. The total digestible nutrients (TDN) indicates the total amount of a meal or diet’s fat, protein, and carbohydrates that are digestible. Energy that can be digested is directly linked to TDN. The TDN is beneficial for cattle cow diets that are mainly foragebased. TDN values frequently underestimate the worth of giving concentrate in comparison to forage. Conversion and processing of food wastes not only prevents waste putrefaction but also helps to conserve abandoned food resources and transform them into economically viable goods [13]. Conversion and processing of FWs avoid putrefaction of wastes. Table 3 outlines several types of FW and the various species of animals that can benefit from its consumption as feed.

### 8.1. Poultry Feed

Poultry is an important component of livestock. Chickens are in such high demand because they are robust animals that mature very quickly in the majority of the world’s regions. Many people throughout the world rely heavily on chicken meat and eggs as a source of protein, as seen by the 25.2 million metric tons (MMTs) of chicken produced in the US alone in 2017 and the expected 83.9 MMTs of broiler output globally in 2018. By 2050, it is anticipated that worldwide meat production willincrease by 66% and that of industrialized nations will increase by 78% [161].

In broiler and layer diets, it is crucial to maintain a balance of nutrients including energy, crude protein, and crude fibre. Analyzing the nutrient makeup of chicken diets is necessary. A well-designed diet based on the nutritional needs of the animal and nutrient analyses of the feed ingredients may boost the animals’ feed conversion ratio. According to numerous studies, when compared with a conventional maize and soy diet, broilers fed food waste consisting of trash from various foods supply chain segments at different percentages performed noticeably similarly. The manufacturing and processing industry produces meat meal, cornflake leftovers, carrot top hay, and dried tomato pomace. Broiler feed has been successfully formulated from bakery trash. Truong et al. [56] discovered that 56-day-old broilers fed only corn/soy had no significant variations in body weights or feed conversion ratios when dried bakery goods up to 10% was added. Additionally, Siddiqui et al. [162] discovered that when compared with 42-day-old broilers fed only corn/soy, the addition of up to 30% dried bakery waste did not significantly alter body weight, feed conversion ratio, or feed intake. When broilers were given beef meal at 65 and 80 g/kg feed in a corn/soy-based diet, they had equivalent daily weight gain, daily feed intake, and feed conversion ratio to birds fed with a full corn/soy diet [163].

### 8.2. Fish Feed

Fish meal is the most valuable commodity in fish farming. Soybean hull, barley, corn, wheat, and other by-products can be used to replace more expensive feed ingredients [164]. Utilizing the by-products of industries that process mangoes allows for the production of freshwater fishes such ascarp and rohu. Fish wastes, including bones, heads, and intestines, as well as FWs such asgroundnut cake, palm kernel cake, wheat bran, rice bran, maize bran, and calf blood, can be used in fish feed production. FW fish feed is generally composed of fruit wastes such as peels with some fruit flesh from pineapple, watermelon, cantaloupe, blackberry, banana, and apple, as well as vegetable wastes such aslettuce, spinach, and so on. Fish feed consists of cereals such as rice bran, soybean meal, rice grain, and spaghetti. The fish feed that was prepared consisted of 60 to 70 percent animal by-products (beef, pork, and chicken) and 30 to 40 percent fish (salmon, etc.) [165]. Carrots have been utilized with mixed results as a source of natural colours. The cichlid fish (*Cichlasomaseverum*) receives 50 mg/kg of total pigments from a diet comprising carrots, resulting in the fish’s colour [139]. There was an increase in pigmentation in prawns given 10 percent frozen carrot tips. Organic wastes suchspent grain from breweries, palm kernel cake, and groundnut cake are used to make more fish meals. Garlic peel has been reported to have immunostimulant properties in aquaculture and to protect African catfish *Clariasgariepinus* from illness [166]. It also boosted resistance to infection by *Aeromonashydrophila* [167].

### 8.3. Cattle Feed

Large volumes of crop-based biomass produced by contemporary agri-food systems are unsuited for direct human consumption but may be used to feed animals for the production of meat, milk, and eggs. Fundamental problems with satisfying the increasing demand for food availability and justice, as well as the urgent need to reduce the impact of food on climate change, environmental degradation, and unsustainable resource exploitation are at the core of the problem [168]. Strategies that encourage circularity and increase the agri-food systems’ potential for regeneration are crucial in this situation. Indigestible, unpleasant, or unwanted biomass (IUUB), which is often unsuited for direct human consumption, is the principal way that biomass materials escape the food supply chain [169]. These materials frequently still contain high levels of proteins, carbs, and other macro-and micronutrients. Due to farm animals’ natural capacity to digest a range of biomass, these nutrients may be recycled by feeding cattle [170]. Consequently, increasing food production while reducing the strain on resources, the environment, and the climate may be accomplished by maximizing the use of IUUB materials through livestock feeding [171]. FWs or discards from different points in the food supply chains that are often not suitable for human consumption. Over the globe, a staggering 1140 MMTs of agricultural wastes and 600 metric tons (MTs) of industrial by-products are frequently utilized as feedstuffs [172]. Yet, there are still enormous quantities of undiscovered resources available for up-cycling using livestock. This is especially true for FWs/discards, which are now estimated to be between 1300 and 1600 MTs globally and 2500 MTs are expected to rise in the future decades [173] the effectiveness of ensiling to preserve leftover fruits and vegetables. On dairy farms, ensiling is a routinely utilized microbiological technique that serves the specific aim of preserving recently harvested feed crops (around 35%) for lengthy storage and feeding [174].

The most effective method is probably using animal feed since it converts FW into food that is high in protein while using very little infrastructure. The World Wildlife Fund (WWF) estimates that by-products from food production and processing make approximately 30% of the feed given to animals globally. Manufacturers and grocery stores supply the vast bulk of the 10% of extra food in the US that is delivered to feed animals [175,176]. Yet, 14.7 MTs of food waste remain that could be utilized as animal feed [177]. The majority of this is disposed of in landfills, which emit methane. Using waste alternatives might assist in preventing further land conversion for producing feed crops according to the WWF, which claims that all food waste to feed component manufacture had a beneficial influence on land usage.

### 8.4. Swine Feed

Swine may integrate dietary fatty acids into meat [178]. Georganas et al. [178] conducted a trial in which pigs were fed only boiled restaurant garbage (26.59% CP, 7.33% total lipids) without any additional food in comparison with a control group that was fed a typical diet (20.21% CP, 15.67% total lipids). Dried ripe banana peels can make up to 20% of the food of developing pigs and 30% of the diet of rabbits [25]. Pigs are mostly fed scraps from kitchens, such as uneaten food and vegetable peels, in addition to other readily available crops and the by-products of agricultural production. Pigs can be directly fed the scraps of fruit and vegetable products that are discarded at marketplaces. The majority of studies on the effect of feeding pigs waste food has been conducted with animals weighing between 50 and 250 pounds [179]. Pigs that are finished off on food scraps often reach a weight of at least 400 pounds before being slaughtered. Intake estimates range from approximately 8 to 10 lb (asfed) per pig per day for pigs weighing less than 100 to 200 lb or more for pigs weighing more than 250 lb [180]. Domesticated pig production in India is mostly reliant on unprocessed agricultural and household waste for the fulfilment of pig diets, whereas the cost of feeding contributes to around 80% of the total expense associated with pork production across the globe [181].

## 9. Safety Policies

Untreated FW may contain pathogens that cause disease. This was demonstrated by the 2001 outbreak of foot-and-mouth disease in the United Kingdom, which was caused by feeding uncooked food waste to pigs. In the same year, the United Kingdom government prohibited the use of FW in animal feeding, and one year later, the European Union issued a similar prohibition. The prohibition does not apply to FWs that contain no meat, fish, or other animal products [182]. These wastes, however, are limited to specific manufacturing by-products and account for a small proportion of EU food wastes. Appropriate heat treatments can make recovered feeds safe for animals by deactivating potentially harmful bacteria and/or viruses that may be present in these types of feeds; we believe that heat-treated waste should be exempt from the ban. Heat treatments can include prolonged heating to temperatures above 70 °C for more than 30 min in order to ensure the safety of the feed produced. Furthermore, when offering leftover human food to animals, disease issues become less important because human food specifications are generally more stringent than animal feed specifications. Furthermore, heat treatment would alleviate problems that arise after waste generation. Unfortunately, current EU bans restrict recycling FW as animal feed, allowing only 3 million tons of manufacturing food losses to be recovered as animal feed out of the 102.5 million tons of FW produced in the EU each year [183].

## 10. Conclusions and Future Perspectives

All across the world, people often throw away a wide variety of food items. Due to the fact that it includes a lot of nutrients, FW can have a number of negative consequences for the environment if it is not properly disposed of or handled. These include, but are not limited to, greenhouse gas emissions, eutrophication, and acidification. Converting food scraps into useful animal feed is a viable solution that can help reduce environmental damage. Though it is normal practice to use leftovers from human meals to feed livestock, scientifically authorized production methods and certified quality feed production are essential for healthy livestock production in all regions. Diverse technologies have been developed for the safe conversion of FW into various dry and liquid livestock feeds. Animal feed made from FW not only replaces commercial feed but also cuts the cost of livestock production. Turning FW into animal feed is one way that can contribute to the creation of a circular economy as well as the achievement of sustainable development. Nevertheless, certification and quality control require a complete characterization of the many forms of FW both before and after the process of conversion into feed. There is an immediate demand for research and development in technology that can convert wasted food into animal feeds that are healthier and more economically viable as well as other useful products. The techniques that are being developed will make it possible to generate data that will be essential in enabling the commercial inclusion of animal feeds that are derived from waste materials to end users. The incorporation of low-cost FW-derived items into animal diets will, in the future, provide the opportunity to reduce production expenses, which account for a significant portion of overall poultry and swine production costs. These expenses account for the opportunity to reduce production costs. Those active in the business world could have a significant incentive to become involved with the practice of using FW as animal feed if they are guaranteed that the practice is safe and of high quality. Last but not least, recycling food scraps for use in animal feed has the potential to improve both food safety and environmental conditions.

Future research must address multiple crucial issues. First, systematic sample collection and comprehensive nutrient analysis are required to provide more accurate information on the complete nutrient profile of pre-treatment food waste and, more importantly, post-treatment feed products in terms of concentration, variability, and bioavailability of key nutrients. This information is crucial for the incorporation of feeds derived from food waste into the precision feeding routine of modern animal production systems. Second, a quantitative assessment is required to link feed grain replacement with resource and environmental benefits throughout the entire food system, including major resource indices such as land, water, energy, fertilizer, and other agricultural inputs and environmental parameters such as soil erosion, nitrogen losses, and greenhouse gas emissions. The scientific and policymaking communities, as well as the public and private sectors, would all benefit from the new understanding provided by this information. The development of effective interventions to support and promote the adoption of food waste recovery for animal feeding also requires input and feedback from stakeholders, such as feed suppliers, livestock producers, food waste emitters, and consumers. The re-feed strategy’s full range of costs and benefits, as well as any potential broader impacts, must be examined in-depth in economic analyses in order to provide the fundamental framework required for the creation of sound, successful, and path-altering policies.

## Figures and Tables

**Table 1:** Country and households’ food waste.

Country	Study Area	Food Waste (kg/Capita)	Reference
India	Andhra Pradesh, Rajam	58	[34]
Pakistan	Gujranwala	88	[35]
Australia	Nationwide	102	[36]
China	Urban China	150	[37]
Japan	Nationwide	64	[38]
Viet Nam	Da Nang	67	[39]
Israel	Nationwide	105	[35]
Bahrain	Nationwide	132	[40]
Lebanon	Beirut	105	[41]
United States of America	NS	59	[42]
Saudi Arabia	Nationwide	105	[43]
Denmark	NS	79	[35]
Mexico	Nationwide	94	[44]
Germany	NS	75	[45]
Hungary	NS	94	[46]
Italy	NS	67	[47]
Netherlands	NS	50	[48]
Brazil	Nationwide	60	[49]
Nigeria	Sapele	189	[50]
Kenya	Nairobi	99	[35]
South Africa	Nationwide	134	[51]
Spain	NS	78	[52]

NS—Not specific.

**Table 2:** Techniques of analyzing different FWs and their use as animal feed.

Food Waste	Processing Techniques	Waste Amount	Animal Feed	Reference
Grape stems	Single-cell production (SCP)	7.5%	Ruminants	[84,85]
Mango peeling	Dehydration and consolidation using either paddy or corn stalks	7–24%	Broilers	[86]
Restaurant FWs	Composition contains corn, soybean meal, and other dietary supplements	45%	Pigs	[87,88]
Pulp from a citrus fruit	The process involves drying and then composing the paddy or corn stalks.	10 million MT of waste each year	Milk-producing cows	[89,90,91,92]
Banana peel	Drying and composition with a standard diet	3.5 MT per year	20 percent for growing pigs and 30 percent for rabbits.	[93,94]
Banana leaves	Ensiling with wheat straw (75/25)	31%	Cows and other animals that give milk	[95,96]
Kitchen wastes	Drying/high temperature composting	37%	Pigs	[97,98]
Mango seeds Bread waste Seasonal fruits Pomace of fruits and olives Lemon peel and non-sterilized fish waste	Ethanolic extract Solid-state fermentation Ruminal fermentation Fermentation Fermentation	42%	Broiler chickens Pigs Cows and other animals that give milk Milk-producing cows Broilers	[99,100,101,102,103,104]

**Table 3:** Types of FWs/by-products and their uses as animal feed.

Food Wastes	Constituent	Animals That Consume It	Reference
Potato waste	Similar to that of corn and barley in terms of energy Crude protein (CP): 7.6%, Ether extract (EE): 7.0%, Crude fibre (CF): 4.0%	Excellent source of energy for cattle feed, 10% to 20% as feed pellets; also used for pigs and goats	[138,139]
Banana root bulbs	Excellent supply of carbohydrates CP: 12.0%, Total digestible nutrients (TDN): 50.0%	Adult cattle can be fed 20–25 kg per day after cleaning and for pig feeding	[139,140]
Apple waste	CP: 12.0%, TDN: 60.0%	30% of this trash can completely replace corn in the feed of poultry and cattle after being chopped, ground, and dried	[139,141]
Rice husk	CF: 39.0–42.0%, EE: 0.8–1.2%, CP: 2.9–3.6%	Cows, horses, and buffaloes	[139,142]
Oil cakes	Vitamin-B- and protein-rich food	Cows, goats, and horses	[139,143]
Barley by-products	Protein 27.0–30.0%, TDN: 65.0%	Dairy cows	[139,144]
Citrus by-products: citrus peel, pulp, rag, seeds	Total sugar (TS): 10.2–16.5%, Crude fat: 1.2–2.2%, CP: 2.2–4.2%, CF: 5.7–8.6%, Nitrogen-free extract 65.0–75.0%	Adult cows 10 kg/day, up to 45% of the main source of energy for beef and other cattle	[139,145,146]
Tea waste	TDN: 58%, CP: 17.94%, Tannic acid: 1.9%	10–15% mixed with a tasty component are fed to cattle	[139,147]
Mango seed kernel	TDN: 55.0%, Protein 6%	20 to 40% for growing calves and buffaloes, 10% for milch cattle, 50% for ruminants, and also as fish feed	[139,148]
Coconut meal	TDN: 70.0–75.0%, CF: 10.0%, CP: 25.0–30.0%	Dairy cows can benefit from a highly helpful protein supplement that boosts milk fat content; also used for goat.	[139,149,150]
Carrot waste	TDN: 75.0–80.0%, Protein 10.0–15.0%, Rich in vitamin A	For cattle, 20 kg/day	[138,139]
Rice bran de-oiled	TDN: 55.0–65.0%, CP: 13.0–16.0%, excellent source of protein, minerals, carbohydrate, vitamins, and high phosphorus content (1.3%)	Cattle, pigs, broiler, fish, and ruminants	[139,151,152]
Jackfruit waste	CP: 7.9%, CF: 14.1%, Calcium (Ca): 0.8%, Phosphorus (P): 0.1%	Cattle, goats, etc.	[138,139]
Tomato waste	TDN: 55.0%, CP: 15.0%	For adult cows up to 50%, and for milch cows and poultry up to 16%	[138,139]
Tamarind seedpowder	TDN: 64.0%, CP: 12.0%	Cattle, broilers, and bullocks	[139,153]
Groundnut meal	TDN: 75.0–85.0%, Protein 40.0–50.0%, High fibre content	Cattle, goats, buffaloes, sheep, and pigs	[139,154]
Citrus molasses	TDN: 65.0–75.0%, CP: 10.0–14.0%, Sugar content 41.0–43.0%	5–10% in the diets of broiler chickens and ruminant feed	[139,155]
Wheat bran	TDN: 65.0–70.0%, CP: 13.0–16.0%, High phosphorus content	Cows, pigs, and goats	[139,156]
Tapioca waste	TDN: 60.0–65.0%, CP: 8.0–12.0%	In order to maintain cattle body weight, 30% of tapioca waste can be fed to adult cattle.	[139,157]
Coffee husk	CP: 7.0–8.0%, Ca: 0.51%, P: 0.25%	Cattle	[139,158]
Soybean meal	TDN: 75.0–84.0%, CP: 45.0–55.0%, Rich in Ca and P	Livestock animal and cattle	[139,159]
Beet molasses	TDN: 65.0–75.0%, CP: 6.0–10.0%	Cows and buffaloes	[139,160]

## Data Availability

Data are included in the article.
